# Trends, key contributors, and emerging issues in honey and breast cancer: A bibliometric analysis from 2014 to 2024

**DOI:** 10.12688/f1000research.159595.2

**Published:** 2025-01-20

**Authors:** Andi Nilawati Usman, Fendi Fendi, Zafitri Nulandari, Dinah Inrawati Agustin

**Affiliations:** 1 Department of Midwifery, Graduate School, Hasanuddin University, Makassar, South Sulawesi, 90245, Indonesia; 2 Research Institute and Community Service, Wuna Agricultural Sciences University, Muna, Southeast Sulawesi, 93654, Indonesia

**Keywords:** honey, breast cancer, ScienceDirect, bibliometric analysis, VOSviewer

## Abstract

**Background:**

Honey, a natural product with diverse bioactive compounds, has been increasingly explored for its potential anticancer properties. This study aims to comprehensively analyze the scientific literature on the relationship between honey and breast cancer.

**Methods:**

A bibliometric analysis was conducted using the ScienceDirect database to identify publications from 2014 to 2024. Data on publication trends, author collaboration, and keyword analysis were extracted to gain insight into the research landscape. Keyword analysis identified nine distinct clusters, indicating diverse research directions regarding the role of honey in breast cancer treatment.

**Results:**

Key journals such as the Journal of Ethnopharmacology and Food Chemistry have been at the forefront of disseminating research findings in this domain, demonstrating a strong interdisciplinary approach that bridges traditional medicine and modern scientific inquiry. The increasing interest in the anticancer properties of honey, as evidenced by the growing number of studies, underlines its potential as a promising natural agent for breast cancer prevention and treatment. Recent advances in the synthesis and theranostic paradigms of cerium oxide nanoparticles (CeONPs) have been highlighted, as well as the potential for selenium nanoconstructs. The toxicity and quality control of Perillae Fructus have also been highlighted.

**Conclusions:**

This study provides a comprehensive overview of the current state of knowledge and uncovers emerging issues that require further investigation.

## Introduction

Breast cancer is the type of cancer that most frequently affects women and has a rather high death rate. Particularly in developing nations, the prevalence rate tends to rise annually, which frequently leads to deaths from delayed identification and treatment, which frequently detects the disease in its terminal stages. ^1^ The malignant growth known as breast cancer (carcinoma mammae) develops in the breast tissue. This cancer starts to spread across the breast’s connective tissue, fatty tissue, and mammary glands. ^2^

Breast cancer is the most often diagnosed disease and the fifth largest cause of cancer mortality worldwide, with a projected 2.3 million diagnoses and 685,000 deaths in 2020, rising to 4.4 million by 2070. Breast cancer accounts for roughly 24.5% of all cancer cases and 15.5% of cancer deaths in women, making it the leading cause of death and incidence in the majority of countries by 2020. ^3^ Approximately half of all breast cancers arise in women who have no known risk factors other than gender and age. Breast cancer will be the most frequent cancer among women in 157 of 185 nations by 2022. Breast cancer affects every country in the world. Men account for around 0.5-1% of all breast cancer cases. ^4^

One of the reasons for the increasing number of cancer cases in Indonesia is that environmental circumstances continue to create harmful substances, such as air, soil, and air pollution, as well as the effects of cigarettes and fast food. ^5, 6^ Other factors that influence this include sleeping too late, a lack of physical activity, and consuming fast food. ^7– 9^

In recent years, numerous items made from different plants, consumables (including fruits, vegetables, and herbs), marine life, and microorganisms have been discovered to be effective in treating a range of human illnesses. ^10^ It has been demonstrated that the efficacy of conventional therapy is increased when they are combined with natural items. ^11^

Traditionally, honey has been used for ages to heal various illnesses. Honey is one of these substances that has gained more attention recently because of its abundance of bioactive chemicals, which are thought to offer several health advantages, including anticancer effects. ^12^ Honey has been discovered to have biological properties such as antioxidant, antibacterial, anticancer, and antiproliferative properties. ^13^ The study of natural substances as potential therapeutic agents in the treatment of cancer has gained significant traction, particularly in the case of breast cancer. ^14^

Bibliometric analysis provides a quantitative and qualitative overview of the scientific literature. ^15^ Through this analysis, we will explore key trends in research by focusing on publications from 2014 to 2024, identify leading contributors in the field, and uncover emerging issues that require further investigation. By systematically examining published research, we seek to provide a comprehensive overview of the current state of knowledge and identify areas that require additional research to fully understand the potential role of honey in breast cancer prevention and treatment.

## Methods

### Bibliometric analysis

Bibliometric analysis is a quantitative methodology that facilitates in-depth mapping, analysis, and interpretation of scientific literature. ^15, 16^ Through this approach, we can identify research trends over time, reveal collaborative networks between researchers, and identify key topics of research focus. Thus, bibliometric analysis provides a comprehensive picture of the research landscape of a field, including the most dominant journals and the most frequently occurring keywords in the related literature.

### Data collection

**Figure 1.**
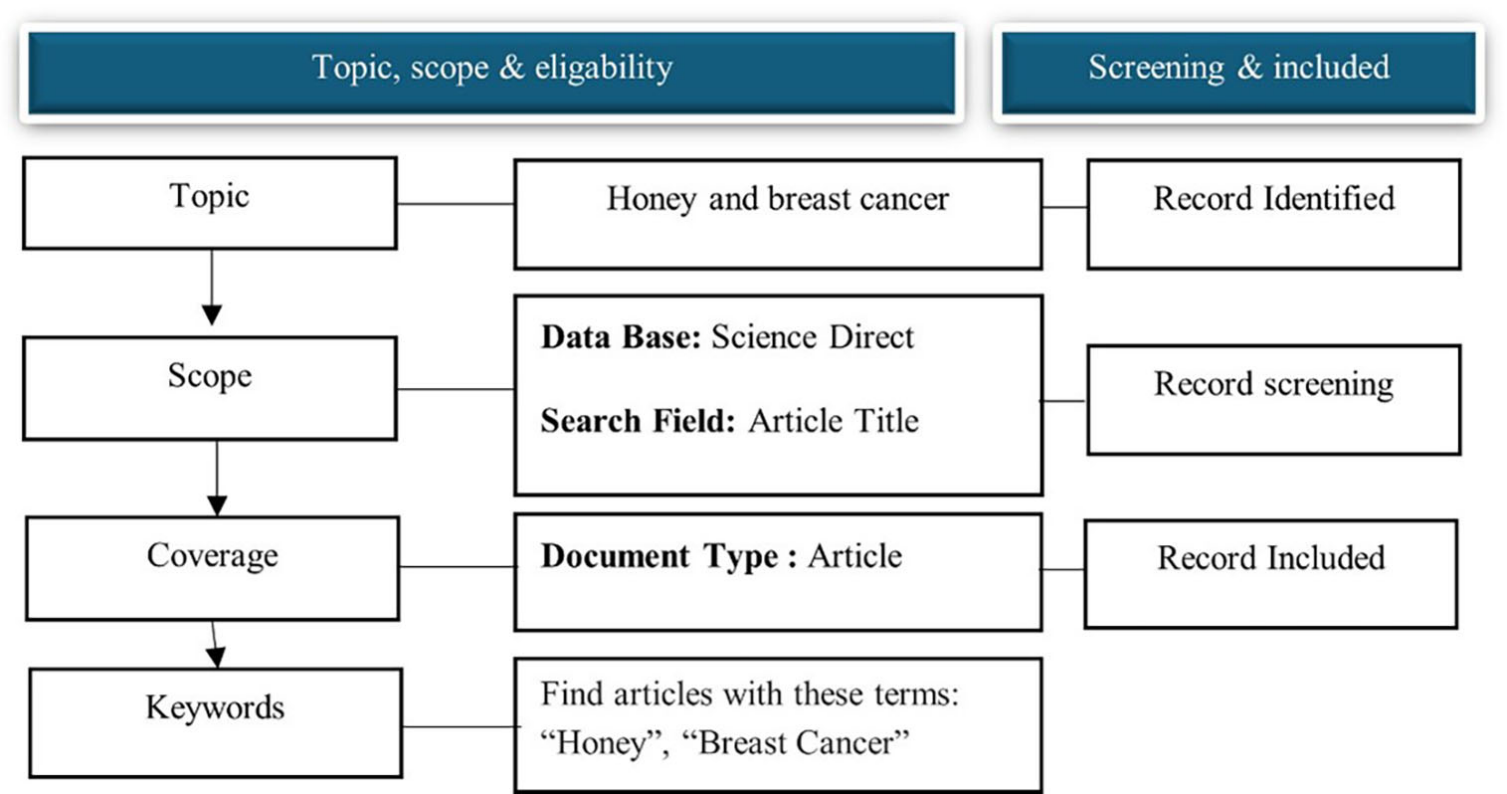
Flow diagram of the search strategy.

Data collection Data was retrieved from the Science Direct database on November 10, 2024. Science Direct was chosen because it is one of the largest searchable databases of abstracts and citations of academic publications compared to other databases, such as Web of Science and Google Scholar, and is continuously expanded and updated. Over 4111 source titles are from over 700 publishers in the Science Direct database. The search terms “honey” and “breast cancer” in the article title were used to search for relevant articles. We refined the search for publication years from 2014 to 2024 to capture current honey and breast cancer research trends. We included published and reviewed articles in English that have appeared in journals, as this visualizes and maps the honey and breast cancer research literature over a decade ( Figure 1).

## Results

### Research results

*Total of publications*

**Figure 2.**
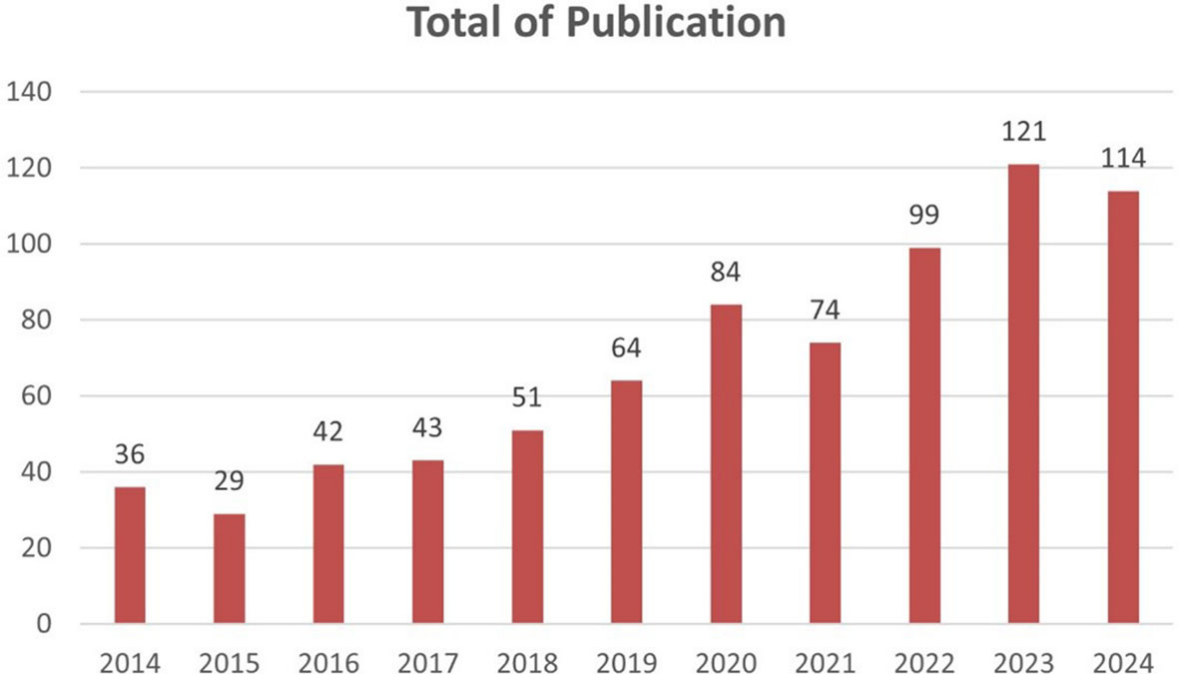
Growth of scientific article publications related to honey and breast cancer published from 2014 to 2024.

Based on Figure 2 there is a significant increase in the number of publications from year to year. Starting from 2014 to 2024, there is a consistent upward trend. The most drastic increase occurred in 2023, with the number of publications reaching 121, an increase of more than three times compared to 2014.

*The journal that publishes most frequently*

The Journal of Ethnopharmacology is the most frequently published journal, with 51 publications. This journal is very relevant because it often publishes research on traditional medicines, including honey, which has potential as an alternative or complementary treatment for various diseases, including cancer. Food Chemistry occupies the second position with several publications 36. This journal is relevant because honey is a natural product with complex chemical content. Research in this field can reveal honey components with antioxidant or antitumor activity. Food Research International is in third place with 17 publications, followed by Food and Chemical Toxicology with 14 publications; both journals focus on food research in general, including functional foods such as honey that have potential health benefits. In seventh place is the Journal of Ayurveda and Integrative Medicine, which is particularly relevant as Ayurveda, the traditional Indian system of medicine, often uses honey to treat various diseases, including cancer. This can also be seen in Table 1.

**Table 1.** The most frequently published journals on honey and breast cancer from 2014 to 2024.

Journal	Total number of issues
Journal of Ethnopharmacology	51
Food Chemistry	36
Food Research International	17
Food and Chemical Toxicology	14
Heliyon	14
Food Bioscience	12
Journal of Ayurveda and Integrative Medicine	11
Journal of Drug Delivery Science and Technology	11
International Journal of Biological Macromolecules	10
LWT	10
Phytomedicine	10
Journal of Food Composition and Analysis	9
Complementary Therapies in Medicine	8
European Journal of Integrative Medicine	8
Journal of Functional Foods	8
Journal of Hazardous Materials	8
Talanta	8
Biomedicine & Pharmacotherapy	7
Environmental Research	7

Based on the data presented in Table 1, the Journal of Ethnopharmacology emerged as the most productive journal in publishing research related to traditional and natural medicines such as honey and breast cancer. This indicates that ethnopharmacology is undergoing rapid development and attracting the interest of many researchers.

In addition, the high number of publications in food-related journals, such as Food Chemistry and Food Research International, indicates that research in the field of food is also very active.

*Co-authorship*

Based on Figure 4, authors Battino, Maurizio, and Giampieri, Francesca appear to be two authors with a total link strength of 44. Their high link strength indicates that they have produced many honey and breast cancer publications and have a wide collaboration network in their research field.

Publishing co-authors who published honey and breast cancer from 2014 to 2024 resulted in 4514 authors. We removed articles with more than 4000 co-authors to avoid considering negligible contributions in the network map. By applying a minimum threshold of 2 published articles per author, 30 authors were identified. Still, only 10 authors were visually mapped in the Figure because some were disconnected.

**Figure 3.**
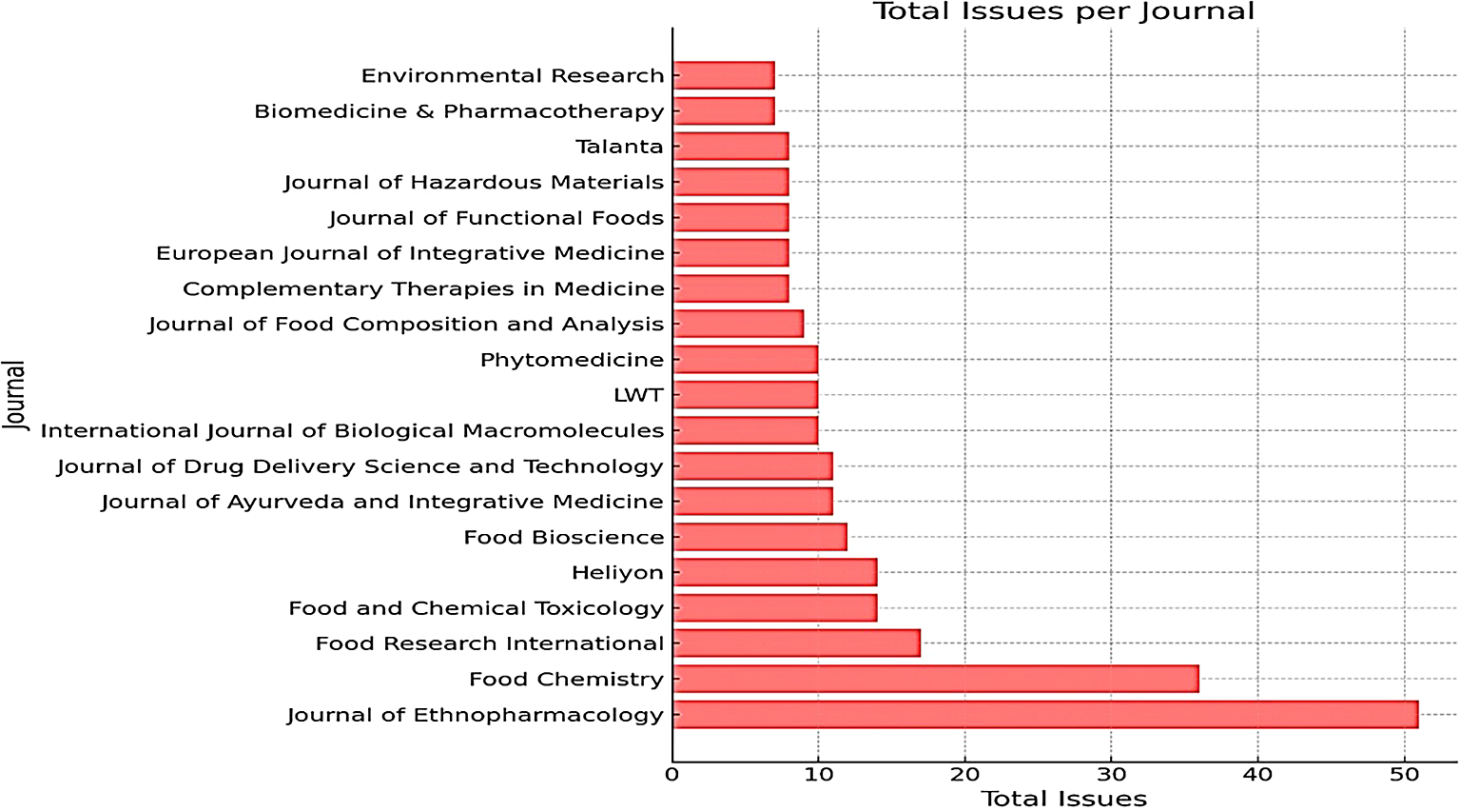
Growth of journals most frequently publishing honey and breast cancer from 2014 to 2024.

**Figure 4.**
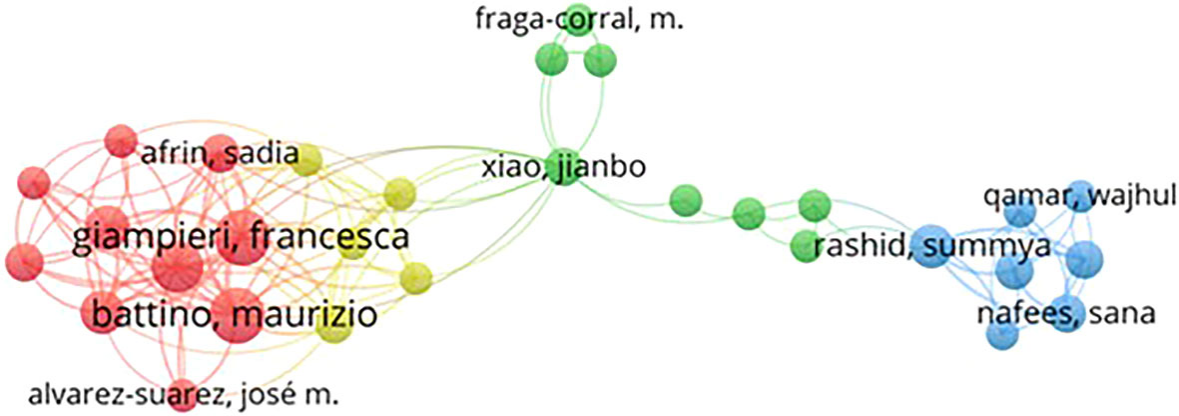
Co-authorship network map of authors publishing on honey and breast cancer from 2014 to 2024.

In Figure 4, the lines between authors represent their collaborative relationships, while 3 different colors represent the authors’ collaboration groups. Although all the top 10 authors listed in Table 2 belong to different groups, their close and strong interconnections indicate a fairly strong research relationship relating to honey and breast cancer. For example, Arifin, Sadia. Alvarez-Suarez, Jose m. Battino, Maurizio. Cianciosi, Danila. Giampieri, Francesca in Cluster 1 (Red), Xiao, Jianbo. Rashid, Summya. Fraga-corral, m. (Green), and Nafees, Sana. Qamar and Wajhul in Cluster 3 (Blue) are closely interlinked, as shown in Figure 3.

**Table 2.** Top 10 authors publishing on honey and breast cancer (rank based on total link strength).

Label	Total Link Strength	Links	Documents	Cluster
Arifin, Sadia	24	14	3	1
Alvarez-suarez, jose m	9	6	2	1
Battino, Maurizio	44	15	7	1
Cianciosi, Danila	39	15	6	1
Giampieri, Francesca	44	15	7	1
Xiao, Jianbo	14	14	3	2
Rashid, Summya	17	9	4	2
Fraga-corral, m.	5	3	2	2
Nafees, Sana	14	6	3	3
Qamar, wajhul	5	5	2	3

### Most frequently occurring keywords

The network appears dense and interconnected, with several clusters forming around the center. This suggests a complex relationship between honey and breast cancer, with many factors contributing to the overall understanding. This co-occurrence network map provides a valuable overview of the current research landscape on honey and breast cancer. The map highlights the potential of honey as a natural remedy for cancer prevention and treatment. The Table 3 shows the Key Clusters and Connections.

**Table 3.** Key clusters and connections.

Cluster	Keywords (Color)	Connections	Reference
**Cluster 1**	**Traditional Knowledge and Ethnobotany (Yellow and Green)**	• **Traditional knowledge:** This cluster highlights the importance of traditional knowledge in understanding and utilizing plants for medicinal purposes. It connects to ethnobotany, medicinal plants, and traditional medicine. • **Ethnobotany:** This cluster focuses on the scientific study of traditional knowledge related to plants, particularly their use in medicine. It links to medicinal plants, human health, ethnomedicine, and ethnopharmacology. • **Medicinal plants:** This cluster represents the core focus of the map, emphasizing the role of plants in traditional and modern medicine. It connects to traditional knowledge, ethnobotany, human health, and ethnomedicine. • **Traditional medicine:** This cluster highlights the use of traditional practices and remedies in healthcare. It connects to medicinal plants, human health, and ethnomedicine.	^17 – 21^
**Cluster 2**	**Human Health and Cancer (Purple and Pink)**	• **Human health:** This cluster emphasizes the broader context of human health, including the role of plants in preventing and treating diseases. It connects to medicinal plants, ethnomedicine, ethnopharmacology, and cancer. • **Cancer:** This cluster focuses on cancer, a major health concern. It connects to human health, ethnomedicine, ethnopharmacology, and various plant-based compounds with potential anticancer properties. • **Ethnomedicine:** This cluster explores the use of traditional medical practices and remedies in different cultures. It connects to human health, cancer, and medicinal plants. • **Ethnopharmacology:** This cluster focuses on the scientific study of traditional medicine and its potential applications in modern healthcare. It connects to human health, cancer, and medicinal plants.	^22 – 27^
**Cluster 3**	**Plant-Based Compounds and their Potential (Blue and Orange)**	• **Quercetin, Pyrrolizidine alkaloids, Rutin, Flavonoids:** These clusters represent various plant-based compounds with potential health benefits, particularly in cancer prevention and treatment. They connect to cancer, antioxidants, inflammation, and apoptosis. • **Antioxidant, Inflammation, Apoptosis:** These clusters represent biological processes targeted by plant-based compounds. They connect to cancer and various plant-based compounds. • **Honey, Food safety, Heterocyclic amines:** This cluster highlights the potential of honey as a food source and its safety concerns related to contaminants. It connects to food safety and heterocyclic amines.	^28 – 32^

**Figure 5.**
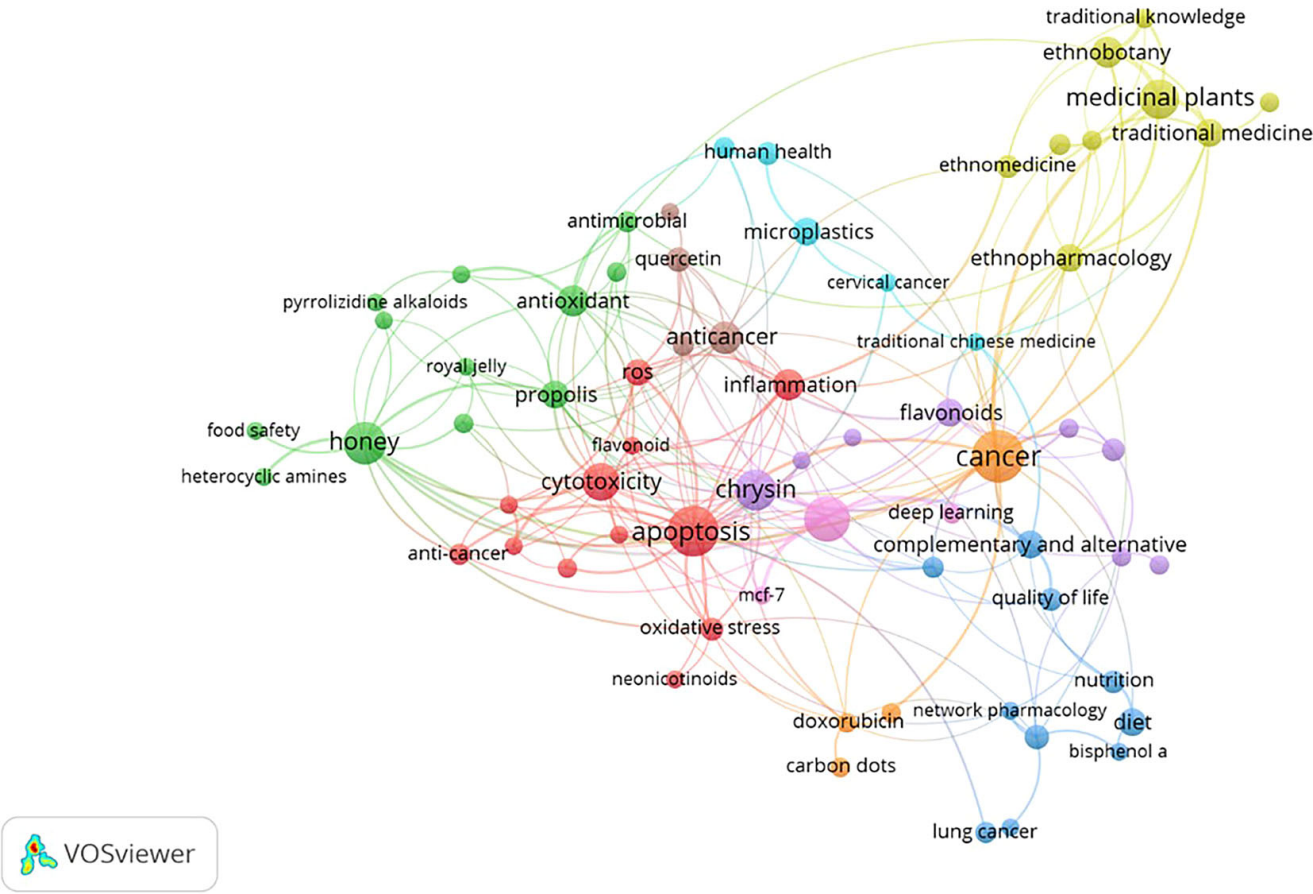
Co-occurrence network map of keywords from articles published on honey and breast cancer from 2014 to 2024.

The co-occurrence analysis of author keywords was conducted from 2014 to 2024 ( Table 4). For the analysis, a threshold of a minimum number of 5 keyword occurrences was set. The analysis yielded 2818 keywords out of 68. The results showed 9 distinct clusters ( Figure 5). Each cluster or combination of clusters represents a subfield of honey and breast cancer research. In particular, as shown in the green, yellow, red, blue, and light blue clusters, keywords such as “Anti-cancer,” “Apoptosis,” and “Inflammation.” “Oxidative stress” is related to Honey as a Cancer Agent. Next, in the green cluster, there are keywords such as “Anti-inflammatory,” “Antimicrobial,” “Antioxidant,” “Honey,” and “Propolis,” which are associated with the antimicrobial effects of honey. in the light blue cluster, the respective keywords are related to the nature of cancer treatment. in the yellow cluster, the respective keys are related to the types of treatment. in the light blue cluster, the respective keys are related to drug development.

**Table 4.** The top 30 keywords of the honey and breast cancer research publication (rank based on total link strength).

Label	Total link strength	Links	Occurrences	Cluster
Anti-cancer	4	5	7	1
Apoptosis	26	24	37	1
Inflammation	9	13	14	1
Oxidative stress	7	10	8	1
Anti-inflammatory	4	5	5	2
Antimicrobial	5	8	7	2
Antioxidant	10	15	14	2
Honey	16	15	26	2
Propolis	9	15	11	2
Antibacterial activity	2	2	5	3
Chemotherapy	5	10	7	3
Quality of life	3	4	8	3
Lung cancer	2	2	7	3
Ethnopharmacology	9	11	11	4
Traditional healers	3	5	7	4
Traditional medicine	10	7	12	4
Bioavailability	2	3	5	5
Pharmacology	1	1	6	5
Phytochemicals	5	7	6	5
Cervical cancer	3	3	5	6
Human health	5	3	8	6
Toxicity	5	5	7	6
Microplastics	4	4	12	6
Drug delivery	3	2	6	7
Cancer	24	25	39	7
Carbon dots	1	1	6	7
Antibacterial	5	6	7	8
Anticancer	10	14	15	8
Breast cancer	20	15	31	9

This visualization presents a comprehensive overview of research topics related to natural products and their health benefits. The network structure, with nodes representing keywords and edges indicating co-occurrence, reveals the interconnectedness of these concepts. Overall, the VOSviewer visualization provides a valuable overview of current research trends in natural products and their health benefits. It highlights the potential of these compounds to address various health challenges and underlines the need for further research to unlock their full potential ( Table 5).

**Table 5.** The VOSviewer visualizations provide a valuable overview of research trends, potential future directions in natural products, and their health benefits.

No	Analyzing Research Trends and Future Innovations	Key Research Trends	Reference
1	**Core Themes and Interconnections**	**Natural Products and Health:** The central theme is the intersection of natural products (honey, propolis, medicinal plants) with human health, particularly in cancer, inflammation, and oxidative stress.	^33 – 38^
		**Traditional Knowledge and Modern Science:** The visualization highlights integrating traditional knowledge (ethnobotany, ethnomedicine) with modern scientific techniques (pharmacology, network pharmacology).	^39 – 44^
		**Bioactive Compounds and Mechanisms:** The visualization emphasizes the role of specific bioactive compounds (quercetin, chrysin, flavonoids) and their mechanisms of action (antioxidant, anti-inflammatory, anticancer).	^45 – 52^
2	**Emerging Trends and Future Directions**	**Nanotechnology and Drug Delivery:** The presence of terms like “carbon dots” and “drug delivery” suggests a growing interest in nanotechnology-based approaches to enhance the delivery and efficacy of natural products.	^53 – 55^
		**Artificial Intelligence and Machine Learning:** Including “deep learning” and “network pharmacology” indicates the potential for AI-driven drug discovery and personalized medicine.	^56 – 60^
		**Environmental Impact and Sustainability:** Terms like “microplastics” and “food safety” highlight the need for sustainable practices in producing and using natural products.	^61 – 64^
3	**Potential Research Gaps and Opportunities**	**Clinical Trials:** While the visualization showcases promising preclinical studies, there is a need for more rigorous clinical trials to validate the efficacy and safety of natural products.	^65 – 68^
		**Mechanistic Studies:** Further research is required to elucidate the precise mechanisms of action of bioactive compounds, particularly at the molecular level.	^69 – 72^
		**Standardization and Quality Control:** Developing standardized methods for the production and quality control of natural products is crucial to ensure consistency and efficacy.	^73 – 76^
		**Ethical Considerations:** Addressing ethical concerns related to the sustainable harvesting of plant resources and the potential for misuse of traditional knowledge is essential.	^77 – 80^
4	**Future Innovations**	**Personalized Medicine:** Utilizing AI and machine learning to identify individuals who may benefit most from specific natural product interventions.	^81 – 84^
		**Combination Therapies:** Developing synergistic combinations of natural products and conventional drugs to enhance therapeutic efficacy.	^85 – 87^
		**Nanotechnology-Based Drug Delivery Systems:** Creating targeted drug delivery systems to improve natural products' bioavailability and therapeutic index.	^88 – 90^
		**Synthetic Biology:** Engineering microorganisms to produce valuable bioactive compounds more efficiently and sustainably.	^91 – 93^

The following table describes the VOSviewer visualization, which provides a valuable overview of research trends and potential future directions in natural products and their health benefits.

## Discussion

The findings indicate a robust interest in exploring honey as a complementary treatment for breast cancer. The increase in publications suggests that researchers increasingly recognize honey’s potential benefits due to its antioxidant and anti-inflammatory properties. The dominance of specific journals indicates a focused interest in ethnopharmacology and food science disciplines. Collaborative networks among top authors demonstrate an active community engaged in this research area, which may lead to more comprehensive studies and innovative therapeutic strategies.

The last decade has witnessed significant evolution in cancer research, particularly in the exploration of natural products as potential therapeutic agents. ^94^ The rich biodiversity in nature provides a variety of unique chemical compounds with promising biological activities. ^95^ In addition, the long history of the use of medicinal plants and the assumption that natural products are safer with fewer side effects further encourage research. ^96^ The active compounds in natural products also serve as inspiration for the development of new synthetic drugs while supporting the principle of cessation. ^97^ Thus, exploring natural products offers new hope in the pharmaceutical world. Among others, honey has attracted attention due to its rich composition of bioactive compounds, which are believed to provide a variety of health benefits. ^98^ According to a growing body of research, honey is not cytotoxic to normal cells but is highly and particularly cytotoxic to tumor cells, suggesting that honey may exhibit anticancer effects. ^99^ From 2014 to 2024, the scientific community is increasingly focused on understanding the essence of honey in breast cancer treatment, reflecting a broader trend towards integrating traditional medicine with modern medical practice.

In 2014, research on honey and its effects on cancer was still emerging, with only a handful of studies investigating its therapeutic potential. However, as awareness of the adverse side effects associated with conventional cancer treatments grew, so did interest in alternative therapies. In 2023, this interest culminated in a marked surge in publications, indicating a paradigm shift toward exploring natural substances such as honey as adjuncts to conventional cancer therapies.

This analytical period highlights the increase in published research and the diversification of study methodologies and interdisciplinary collaborations. Researchers from diverse fields-from pharmacology to nutrition, have begun to converge on this topic, fostering a rich environment for innovation and discovery. This ten-year analysis of publications reveals key trends in authorship, journal contributions, and thematic focus areas, providing insight into how Honey is positioned within the broader context of breast cancer research. ^100– 102^ As we delve deeper into this analysis, it becomes clear that the intersection of traditional knowledge and scientific inquiry is paving the way for new therapeutic approaches. ^103^ The growing body of literature underscores honey’s potential role as a valuable resource in breast cancer treatment strategies and emphasizes the need for continued research to elucidate its mechanisms and efficacy fully. ^104, 105^

The period from 2014 to 2024 has witnessed a tremendous increase in publications related to honey and breast cancer. Initially, research in this field was still limited, but a significant surge in interest became apparent in 2023 when the number of publications jumped to 121- more than three times the 2014 results. the 2014 results trend reflects the growing recognition of the potential therapeutic properties of honey, including its antioxidant and anti-inflammatory effects, which are particularly important in the fight against cancer. This analysis highlights that key journals such as the Journal of Ethnopharmacology and Food Chemistry have been at the forefront of disseminating findings in this domain, indicating the involvement of the findings in this domain and indicating a strong engagement from ethnopharmacology and food science perspectives.

The co-authorship network identifies key contributors to this field, highlighting leading researchers such as Maurizio Battino and Francesca Giampieri. Their collaboration and extensive publication record underscore the importance of collective efforts in advancing knowledge on the therapeutic effects of honey. The analysis also reveals that leading journals such as the Journal of Ethnopharmacology and Food Chemistry have played an important role in disseminating research findings, demonstrating a strong interdisciplinary approach that bridges traditional medicine and modern scientific inquiry.

Furthermore, this bibliometric analysis investigates emerging issues in the research landscape, identifying important themes through keyword co-occurrence analysis. Key terms such as “anticancer,” “apoptosis,” “inflammation,” and “oxidative stress” reflect the multifaceted nature of honey’s role in cancer treatment. Identifying different research groups suggests that research increasingly focuses on different aspects of honey’s biological activity, including its antimicrobial properties and potential applications in drug development.

The limitation of this study is that it still does not explore clinical trials enough and needs further research. Clinical trials are essential in bridging the gap between preclinical findings and broader clinical implementation. Most findings to date have come from in vitro studies or animal models. Human clinical trials are needed to ensure that honey is not only effective but also safe to use as an adjunct therapy in the context of breast cancer. This includes assessing appropriate dosage, potential side effects, and interactions with other therapies such as chemotherapy or radiotherapy.

Honey contains various bioactive compounds with antioxidant, anti-inflammatory, and anti-cancer activities. Clinical trials can help determine the most effective formulation of honey and the optimal method of administration, such as oral, topical, or combination. This standardization is essential to ensure consistency of clinical outcomes.

Clinical trials can also evaluate honey’s benefits in improving cancer patients’ quality of life. The focus may include honey’s effects on reducing fatigue and nausea and other side effects of conventional therapies. This research is important for integrating honey into a holistic cancer care approach.

## Conclusion

This bibliometric analysis highlights the growing interest in honey as a potential adjunctive therapy for breast cancer. The increasing number of publications indicates a promising future for honey research. Moreover, this analysis emphasizes the potential of plant-based compounds, especially those derived from honey, as promising therapeutic agents for cancer. Integrating traditional knowledge with modern scientific methods is essential to unlock the full potential of these compounds. Further research is needed to elucidate their mechanism of action, assess their safety and efficacy, and develop evidence-based interventions. By bridging the gap between traditional knowledge and scientific research, we can harness the power of nature to improve human health.

## Author contributions

ANU, FF, ZN, and DIA contributed to the literature review, data extraction from various databases, conceptualization, development of the economic models on Microsoft Excel Software, formal analysis, findings interpretation, and manuscript writing. All authors approved the final version of the paper.

## Ethic and consent

Ethical approval and consent were not required.

## Data Availability

Figshare: **Trends, key contributors, and emerging issues in honey and breast cancer: A bibliometric analysis from 2014 to 2024**, Doi: https://doi.org/10.6084/m9.figshare.28013666. ^106^ The project contains the following underlying data:
- data figshare_bibliomettik_F1000.xlsx (This study examined the relationship between honey and breast cancer. The researchers analyzed scientific publications from 2014 to 2024 to understand research trends, researcher collaborations, and key topics studied). Data are available under the terms of the Creative Commons Attribution 4.0 International license (CC-BY 4.0). Figshare: PRISMA 2020 checklist for the article “Trends, key contributors, and emerging issues in honey and breast cancer: A bibliometric analysis from 2014 to 2024” has been uploaded to Figshare and is available at the following DOI: https://doi.org/10.6084/m9.figshare.28041074. ^107^ Data are available under the terms of the Creative Commons Attribution 4.0 International license (CC-BY 4.0).
