# Supplementary material for: Trends, key contributors, and emerging issues in honey and breast cancer: A bibliometric analysis from 2014 to 2024
Source: F1000Res. 2025 Mar 24;14:17. Originally published 2025 Jan 3. [Version 3] doi: 10.12688/f1000research.159595.3 (PMC11983675; doi:10.12688/f1000research.159595.3)
Supplement: Peer review history

# Reviewer report: Reviewer response for version 3

**DOI:** 10.5256/f1000research.179237.r372708

**Published:** 2025-04-07

**Authors:** Riadh Badraoui

**Affiliations:** 1 The University of El Manar (UTM), Tunis, Tunisia

The manuscript has been improved. Its revised version is acceptable and warrants indexing.

Is the work clearly and accurately presented and does it cite the current literature?

Yes

If applicable, is the statistical analysis and its interpretation appropriate?

Not applicable

Are all the source data underlying the results available to ensure full reproducibility?

Yes

Is the study design appropriate and is the work technically sound?

Yes

Are the conclusions drawn adequately supported by the results?

Yes

Are sufficient details of methods and analysis provided to allow replication by others?

Partly

Reviewer Expertise:

Antioxidants, breast cancer, skeletal metastases, Pharmacokinetics, Molecular interactions, Oxidative injury

I confirm that I have read this submission and believe that I have an appropriate level of expertise to confirm that it is of an acceptable scientific standard.

# Reviewer report: Reviewer response for version 2

**DOI:** 10.5256/f1000research.176942.r368954

**Published:** 2025-03-03

**Authors:** Riadh Badraoui

**Affiliations:** 1 The University of El Manar (UTM), Tunis, Tunisia

Review for F1000Research

 In this original article entitled “ *Trends, key contributors, and emerging issues in honey and breast cancer: A bibliometric analysis from 2014 to 2024*”, the authors (Usman et al.) analyzed the scientific literature on the relationship between honey and breast cancer.

 The manuscript is acceptable but requires some minor to major improvements. Hereafter some comments revealed after reviewing its current version.

1. There should be an indication about the used keywords for the bibliometric study in the section of Methods itself.
2. What are the inclusion and exclusion criteria of the selected studies from 2014 to 2024? Please clarify.
3. We are already in 2025, but the authors used the future while writing about 2020: “Breast cancer will be the … nations by 2022.” Please check throughout the whole manuscript.
4. The relationship between the honey phytochemical composition and the anticancer effect is overlooked. Such focus would have additional value to the manuscript.
5. Both sentences “Breast cancer is the type …high death rate.”, and “Breast cancer is the most often diagnosed d” can be supported by the following relevant and recent references; Badraoui R, et al., 2009 [Ref-1] and Bouali N, et al., 2024 [Ref-2]. Likewise, “The last decade has witnessed…therapeutic agents” can be supported by Badraoui R, et al., 2020 [Ref-3]
6.  English language is overall acceptable but minor editing and checking are required.  

Is the work clearly and accurately presented and does it cite the current literature?

Yes

If applicable, is the statistical analysis and its interpretation appropriate?

Not applicable

Are all the source data underlying the results available to ensure full reproducibility?

Yes

Is the study design appropriate and is the work technically sound?

Yes

Are the conclusions drawn adequately supported by the results?

Yes

Are sufficient details of methods and analysis provided to allow replication by others?

Partly

Reviewer Expertise:

Antioxidants, breast cancer, skeletal metastases, Pharmacokinetics, Molecular interactions, Oxidative injury

I confirm that I have read this submission and believe that I have an appropriate level of expertise to confirm that it is of an acceptable scientific standard, however I have significant reservations, as outlined above.

## References

[1] : Effect of alpha tocopherol acetate in Walker 256/B cells-induced oxidative damage in a rat model of breast cancer skeletal metastases. *Chem Biol Interact* .2009;182(2-3) : 10.1016/j.cbi.2009.09.010 98-105 10.1016/j.cbi.2009.09.010 19781538

[2] : GC-MS screening of the phytochemical composition of Ziziphus honey: ADME properties and in vitro/in silico study of its antimicrobial activity. *J Biomol Struct Dyn* .2024;42(3) : 10.1080/07391102.2023.2205945 1368-1380 10.1080/07391102.2023.2205945 37191027

[3] : Allium subhirsutum L. as a Potential Source of Antioxidant and Anticancer Bioactive Molecules: HR-LCMS Phytochemical Profiling, In Vitro and In Vivo Pharmacological Study. *Antioxidants (Basel)* .2020;9(10) : 10.3390/antiox9101003 10.3390/antiox9101003 33081189 PMC7602730

# Reviewer report: Reviewer response for version 2

**DOI:** 10.5256/f1000research.176942.r360873

**Published:** 2025-01-27

**Authors:** Mogana Das Murtey

**Affiliations:** 1 Universiti Sains Malaysia, Minden Heights, Pulau Pinang, Malaysia

Its good.

Is the work clearly and accurately presented and does it cite the current literature?

Yes

If applicable, is the statistical analysis and its interpretation appropriate?

Yes

Are all the source data underlying the results available to ensure full reproducibility?

Yes

Is the study design appropriate and is the work technically sound?

Yes

Are the conclusions drawn adequately supported by the results?

Yes

Are sufficient details of methods and analysis provided to allow replication by others?

Yes

Reviewer Expertise:

Cancer Biology

I confirm that I have read this submission and believe that I have an appropriate level of expertise to confirm that it is of an acceptable scientific standard.

# Reviewer report: Reviewer response for version 1

**DOI:** 10.5256/f1000research.175350.r355982

**Published:** 2025-01-10

**Authors:** Mogana Das Murtey

**Affiliations:** 1 Universiti Sains Malaysia, Minden Heights, Pulau Pinang, Malaysia

The article is well-structured, provides a thorough bibliometric analysis, and appropriately cites current literature from 2014 to 2024. However, some minor details in the keyword co-occurrence analysis could be expanded for clarity. The methodology, using bibliometric analysis via ScienceDirect, is appropriate and robust. The analysis tools, such as VOSviewer, were suitably employed to map research trends. The methods section provides adequate detail about data collection, inclusion criteria, and analytical tools, enabling replication.

 The bibliometric statistics and visualization interpretations are appropriate and effectively highlight trends and clusters. The authors have deposited the data in a public repository (Figshare) and described it adequately. The conclusions align with the results, highlighting honey's therapeutic potential in breast cancer, but a discussion of clinical trial needs could be elaborated.

Is the work clearly and accurately presented and does it cite the current literature?

Yes

If applicable, is the statistical analysis and its interpretation appropriate?

Yes

Are all the source data underlying the results available to ensure full reproducibility?

Yes

Is the study design appropriate and is the work technically sound?

Yes

Are the conclusions drawn adequately supported by the results?

Yes

Are sufficient details of methods and analysis provided to allow replication by others?

Yes

Reviewer Expertise:

Cancer Biology

I confirm that I have read this submission and believe that I have an appropriate level of expertise to confirm that it is of an acceptable scientific standard.
